# Hospitalizations of the older adults with and without dementia during the last two years of life: the impact of comorbidity and changes from 2002 to 2017

**DOI:** 10.1007/s40520-024-02918-0

**Published:** 2025-01-21

**Authors:** Saritha Susan Vargese, Marja Jylhä, Jani Raitanen, Leena Forma, Mari Aaltonen

**Affiliations:** 1 Faculty of Social Sciences (Health Sciences, Gerontology Research Center (GEREC), Tampere University, Tampere, Finland; 2 Believers Church Medical College Hospital, Pathanamthitta, Kerala India; 3 The UKK Institute for Health Promotion Research, Tampere, Finland; 4 University of Eastern Finland, Kuopio, Finland; 5 Finnish Institute for Health and Welfare, Helsinki, Finland

**Keywords:** Dementia, Comorbidities, Long term care, Hospitalizations

## Abstract

**Background:**

Multimorbidity creates challenges for care and increases health care utilization and costs. People with dementia often have multiple comorbidities, but little is known about the role of these comorbidities in hospitalizations.

**Aims:**

This study examines the frequency of hospitalizations during the last two years of life in older adults with and without dementia, the impact of comorbidities on hospitalizations, and their time trends.

**Methods:**

The data came from national registers and covered all persons 70 and above who died in Finland in 2002–2017. The effect of dementia and comorbidities on hospitalizations in the last two years of life was determined using binary logistic regression and negative binomial regression.

**Results:**

At all levels of comorbidity, people with dementia were less likely to be hospitalized and had a lower number of hospitalizations than people at the same level of comorbidity but no dementia. Hospitalizations were strongly associated with multimorbidity. During the study period, the overall hospitalization rates from home and LTC have declined.

**Discussion:**

The declining trend of hospitalization during the 15-year study period should be interpreted in the context of the health and long-term care system.

**Conclusion:**

Among people with dementia, comorbidities were the main driver for hospitalizations. Regardless of the number of comorbidities, people with dementia were hospitalized less often than people without dementia in last two years of life. It remains unclear whether the lower hospitalization rate is due to the improved ability to care for people with dementia outside the hospital or to the lack of sufficient medical care for them.

**Supplementary Information:**

The online version contains supplementary material available at 10.1007/s40520-024-02918-0.

## Introduction

Hospitalizations at the end of life have been the subject of research interest especially in late-stage dementia as this vulnerable group is at risk for experiencing poorer health and functional outcomes during and post hospitalization [1, 2]. In all, hospitalizations in people with dementia are associated with higher rates of complications, longer hospital stays and costs, readmissions, and mortality compared to those without dementia [3–7].

It is known that dementia, multimorbidity, and old age are all associated with higher hospitalization rates [3, 8–11]. People with dementia typically have 2–8 additional comorbidities and have higher levels of multimorbidity compared to people of similar age but without dementia [12–14]. Earlier evidence implies that both dementia and multimorbidity increase health care utilization, including hospitalizations, and costs of care [1, 3–6, 8–11], but the role of comorbidities in people with dementia in hospitalizations remains unclear.

A recent meta-analysis on hospitalization rates and their predictors in the United States, Europe, and Asia reported that people with dementia were likely to be admitted to the hospital more frequently than those without dementia, regardless of the number of comorbidities [12]. Hospitalization relates to the severity of people’s illness, and therefore, it is not surprising that at the population level, hospitalizations tend to increase towards the end of life [13]. However, studies focusing on hospital use during the last years of life reported less frequent hospitalizations among people with dementia than those without [14–15], suggesting that the frequency of hospitalizations is related to the stage of dementia and closeness of death. In severe dementia, people often live in long-term care facilities [16]. Studies from different European countries report that during their last years of life, 8–51% of residents in long-term care facilities with dementia were hospitalized [2, 17, 18]. These extensive country differences may be attributed to differences in health care and long-term care systems, policy, and the study period.

This study focuses on the association of dementia and comorbidity with hospitalizations in the last two years of life among people aged 70 or over who died in Finland in 2002, 2006, 2010, 2014, or 2017. Using exhaustive nationwide register-based data, we set out to answer three questions: (1) How frequent are hospitalizations in the last two years of life among older adults with dementia compared to those without? (2) What is the joint impact of dementia and the number of comorbidities on hospitalizations? (3) How did the frequency of hospitalizations in the last two years of life among older people with and without dementia change between the study years? This study is part of the ongoing project, The longevity revolution: implications for the need and cost of health and social care (COCTEL) [19].

## Methods

### Data

The study population comprised five cohorts of people in Finland who died at age 70 or above in 2002 (*N* = 35,821), 2006 (*N* = 34,123), 2010 (*N* = 36,895), 2014 (*N* = 39,351), and 2017 (*N* = 41,496). They were identified from the Causes of Death Register and included all individuals who died at this age in Finland in these years.

Morbidity data based on ICD-10 codes for two years before death were extracted from national registers as described below. A person was considered to have the diagnosis if it was indicated in any of the three source registers, namely *The Causes of Death Register* (Statistics Finland), *The Care Register for Health Care*, and *The Care Register for Social Welfare* (Finnish Institute for Health and Welfare). These exhaustive care registers cover all use of hospital care and long-term care services, and information on diagnoses recorded in admission. The Causes of Death Register includes three types of causes of death, immediate, intermediate, and underlying cause of death, and all these were used to identify the diagnoses. The dementia group was identified using ICD-10 codes F00 (dementia in Alzheimer’s disease), F01 (vascular dementia), F02 (dementia in other diseases), F03 (unspecified dementia) or G30 (Alzheimer’s disease). In addition to dementia, other diagnoses included were hypertension (I10–I16), coronary artery disease (CAD) (I20–I25), diabetes (E10–E14), stroke (I60–I69), Parkinson’s disease or other neurological diseases (G00–G99 excluding G30), asthma or chronic obstructive pulmonary disease (COPD) (J40–J47), cancer (C00–C97), hip fracture (S72), psychotic or neurotic disorders (F20–F29, F40–F48), and depression (F32, F33). The selection of morbidities was based on their high prevalence in the older population [20] and their known associations with dementia [21, 22].

### Outcome variables

The outcome of interest was hospitalization, which was considered as (1) at least one hospitalization (0 = no, 1 = yes) and (2) the number of hospitalizations in the last two years of life. Hospital was defined as including any type of hospital, such as university, central, or regional hospital, as well as short-term care periods (< 90 days) at the health center, a health care facility offering primary hospital care. Hospitalizations were analyzed in the total study group and separately for hospitalizations from home to hospital and from LTC to hospital, based on where the individual was living before hospitalization. Long-term care included residential homes, service housing with 24-hour assistance, and the health center ward (length of stay ≥ 90 days). Every movement from home or LTC to hospital was counted as one hospitalization. The variable ‘hospitalizations’ was considered as a count variable.

### Explanatory variables

The main explanatory variable of interest was dementia. In addition, we constructed a variable that combined dementia and the number of comorbidities. Age was considered as a covariate in the main analysis.

### Statistical analysis

Descriptive statistics were used to describe the basic characteristics separately for people with dementia (D + group) and without dementia (D- group) in each study year (year of death). Pearson’s chi-square test was used to examine the association between dementia status and sex (men or women), age (70–79, 80–89, ≥ 90), and the number of morbidities (0–1, 2, 3, ≥4). Independent samples t-test was used to analyze differences in age between those with and without dementia in each study year. Frequencies (%) were calculated for people with at least one hospitalization according to age and sex in each study year. The mean and standard deviation (SD) of hospitalizations were calculated separately for individuals with and without dementia, as well as for hospitalizations from home and long-term care (LTC).

The association of dementia and comorbidities with at least one hospitalization from home or LTC was examined using binary logistic regression. Odds ratios (OR) and their 95% confidence intervals (CI) were obtained from the whole group and age (70–79, 80–89, 90+) and sex separated models. The association of dementia and comorbidities with the number of hospitalizations was determined by using negative binomial regression. Incidence rate ratios (IRR) and their 95% CIs were obtained from the models separately for age groups and sex. The models were adjusted for age in the whole group and sex separated analyses. The number of days at home or LTC (person-time in risk) was included as an offset variable in the negative binomial regression.

We conducted separate analyses for those hospitalized from home and LTC. In a logistic regression model with at least one transition from home or LTC to hospital as the outcome, and age, dementia status, and study year as explanatory variables, an interaction term was added to assess the differences in hospitalizations among people with and without dementia over the study years.

Statistical analyses were performed using Stata 16 (College Station, TX, USA). We considered statistical significance at *p* < 0.05.

## Results

### Descriptive findings

Almost one-third (31.2%) of the study population (*N* = 187,686) had a diagnosis of dementia at the time of death. The figures for 2002, 2006, 2010, 2014, and 2017 were 24.2%, 26.9%, 30.9%, 35.4%, and 37.1% respectively. Among both people with and without dementia, mean age at death increased during the study period. In each study year, the proportion of women and mean age at death were significantly higher for those with dementia. The most common morbidities in people with and without dementia were respiratory diseases, hypertension, coronary artery disease and stroke. In all study years, people with dementia more frequently had three or more morbidities compared to those without dementia (Table 1).

**Table 1:** Basic characteristics of the participants: people 70 years and above who died in 2002, 2006, 2010, 2014, and 2017

Variables	2002		2006		2010		2014		2017	
	D+	D-	D+	D-	D+	D-	D+	D-	D+	D-
n	8700	27,121	9188	24,935	11,416	25,479	13,963	25,388	15,418	26,078
Gender (%)										
Women	69.2	55.9	67.5	53.3	66.6	52.6	65.9	51.6	63.7	49.5
Men	30.8	44.1	32.5	46.7	33.4	47.4	34.1	48.4	36.3	50.5
p^1^	< 0.001		< 0.001		< 0.001		< 0.001		< 0.001	
Age at death (%)										
70–79	18.9	40.3	16.9	37.6	14.0	34.7	12.0	35.3	12.1	37.1
80–89	51.9	42.5	52.2	44.7	53.7	46.2	51.2	43.7	48.5	41.4
≥90	29.2	17.2	30.9	17.7	32.3	19.1	36.9	20.9	39.4	21.5
p^1^	< 0.001		< 0.001		< 0.001		< 0.001		< 0.001	
Age at death, mean (SD)	85.5 (6.4)	82.0 (7.2)	85.9 (6.4)	82.3 (7.2)	86.4 (6.3)	82.8 (7.2)	87.1 (6.2)	82.9 (7.5)	87.4 (6.3)	82.7 (7.7)
p^2^	< 0.001		< 0.001		< 0.001		< 0.001		< 0.001	
Number of morbidities ^#^										
0–1	8.8	26.2	8.6	24.3	10.0	23.1	13.0	21.7	15.1	21.2
2	28.4	32.4	26.3	32.1	25.8	31.6	26.2	30.6	25.5	30.5
3	31.5	25.6	30.6	26.3	29.7	26.8	28.2	27.6	27.4	27.4
≥4	31.3	15.8	34.5	17.3	34.5	18.6	32.6	20.1	31.9	20.9
p^1^	< 0.001		< 0.001		< 0.001		< 0.001		< 0.001	

^1^ Pearson’s chi-square test (people with dementia (D+) vs. without dementia (D-) within a year)

^2^ Independent samples t-test (D + vs. D- within a year)

# Including dementia

SD = standard deviation

### Hospitalizations in people with and without dementia

Hospitalizations, both in the whole group, from home, and from LTC, were less common for people with dementia than for those without in all study years (Table 2). The proportion of men with dementia hospitalized at least once from home varied between 83.7% and 88.3% in the study years, and from LTC between 43.8% and 47.0%. The corresponding proportions for women were 77.2% and 81.8%; and 37.3% and 41.8%, respectively. Mainly, in men and women and in all age groups, those with dementia had fewer hospitalizations than those without dementia. Only among men from 2010 onwards, a slightly higher proportion of those with than without dementia were hospitalized from LTC. In the total group with dementia, hospitalizations were more frequent among men than women and in the youngest group studied. In the group without dementia, differences between sex and age groups were smaller or negligible (Table 2). The mean (SD) of hospitalizations for people with and without dementia were 2.42 (2.98) and 3.30 (3.42) respectively. For hospitalizations from home, the mean (SD) for people with and without dementia were 2.64 (3.12) and 3.28 (3.42) and for those from LTC were 0.80 (1.33) and 0.94 (1.59) respectively.

**Table 2:** Proportion of people with at least one hospitalization among people with (D+) and without (D-) dementia who died in 2002, 2006, 2010, 2014, and 2017

Transition		2002		2006		2010		2014		2017	
		D+	D-	D+	D-	D+	D-	D+	D-	D+	D-
Home/LTC to hospital	Men	80.9	87.7	82.4	88.2	81.2	87.2	80.4	87.1	81.1	88.0
	Women	67.6	86.1	67.9	87.0	68.1	87.1	67.5	87.8	66.6	87.5
	p^1^	< 0.001	< 0.001	< 0.001	0.003	< 0.001	0.837	< 0.001	0.101	< 0.001	0.170
	70–79	80.2	87.1	79.9	87.2	78.2	86.1	75.6	86.2	75.2	86.3
	80–89	74.4	88.4	75.9	88.9	75.2	88.5	75.6	89.1	74.7	89.4
	≥90	61.5	82.2	63.0	85.1	65.4	85.6	65.7	86.1	67.4	87.3
	p^1^	< 0.001	< 0.001	< 0.001	< 0.001	< 0.001	< 0.001	< 0.001	< 0.001	< 0.001	< 0.001
Home to hospital	Men	88.3	88.8	88.5	88.6	85.6	87.4	83.7	87.3	83.9	88.3
	Women	81.8	90.0	81.8	89.6	79.4	88.9	78.5	89.3	77.2	89.2
	p^1^	< 0.001	0.004	< 0.001	0.021	< 0.001	< 0.001	< 0.001	< 0.001	< 0.001	0.030
	70–79	88.6	88.1	87.1	87.8	84.4	86.7	82.1	86.4	80.5	86.7
	80–89	85.6	90.9	85.6	90.3	82.2	89.1	83.0	90.0	81.7	90.4
	≥90	76.8	89.1	79.7	89.1	79.7	88.7	76.0	88.0	77.3	89.2
	p^1^	< 0.001	< 0.001	< 0.001	< 0.001	0.002	< 0.001	< 0.001	< 0.001	< 0.001	< 0.001
LTC to hospital	Men	45.7	47.2	46.6	47.6	47.0	46.7	46.2	42.7	43.8	42.3
	Women	41.8	46.5	41.8	46.7	41.7	46.5	39.5	45.9	37.3	42.0
	p^1^	0.003	0.508	< 0.001	0.415	< 0.001	0.801	< 0.001	0.004	< 0.001	0.824
	70–79	43.9	45.0	42.1	46.3	43.5	43.1	41.1	44.6	40.0	41.1
	80–89	44.8	47.4	46.3	47.4	46.1	47.0	43.5	44.2	41.1	42.5
	≥90	39.1	47.0	38.9	47.1	39.0	48.1	39.5	45.3	37.7	42.3
	p^1^	< 0.001	0.157	< 0.001	0.738	0.002	0.003	< 0.001	0.647	0.001	0.613

^1^ Pearson’s chi-square test (hospitalization vs. sex or hospitalization vs. age within a year and dementia status)

LTC = long-term care

### Joint impact of dementia and comorbidities on hospitalizations in the last two years of life

**Fig. 1:**
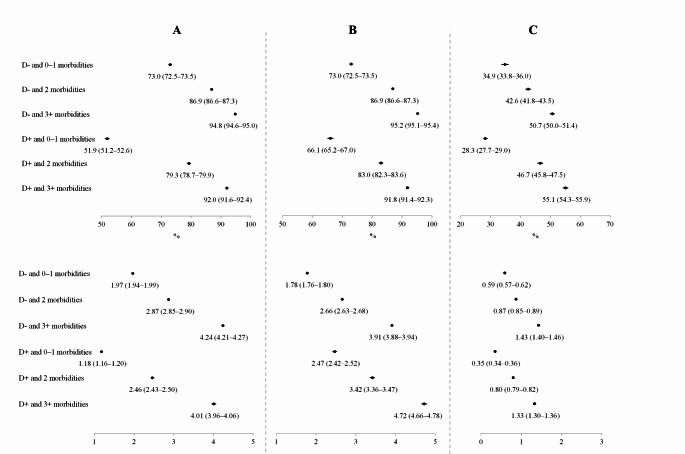
Predicted probabilities of hospitalizations and number of hospitalizations (**a**) from home or LTC, (**b**) from home, and (**c**) from LTC. The upper panel is based on logistic regression models and the lower panel is based on negative regression models

At all levels of comorbidity, people with dementia were less likely to be hospitalized at all than people without dementia. This was true for all age groups and both genders. Hospitalizations from home followed the same pattern. Also, hospitalizations from LTC were more frequent among those without dementia in the group that had only 0–1 other morbidities. At higher levels of comorbidity, hospitalizations were more likely among those with dementia in the whole group, in 80-89-year-olds and for both gender groups, while there were no significant differences in the age groups of 70–79 and 90+ (Table 3). The predicted probabilities of hospitalizations from logistic regression model is shown in Fig. 1 (upper panel).

**Table 3:** Association between dementia and comorbidities with hospitalization from home/LTC, from home, and from LTC (separated models for whole group, age groups, and gender)

	All	70–79	80–89	90+	Women	Men
	OR (95% CI)	OR (95% CI)	OR (95% CI)	OR (95% CI)	OR (95% CI)	OR (95% CI)
**Hospitalization from home or LTC** ^**1**^						
D- and 0–1 morbidities	1	1	1	1	1	1
D- and 2 morbidities	2.46 (2.37–2.56)	2.62 (2.46–2.79)	2.41 (2.27–2.57)	2.12 (1.95–2.31)	2.17 (2.06–2.29)	2.82 (2.66–2.99)
D- and 3 + morbidities	6.75 (6.46–7.06)	7.56 (7.02–8.15)	6.50 (6.06–6.97)	5.48 (4.99–6.03)	5.75 (5.41–6.11)	8.06 (7.54–8.62)
D + and 0–1 morbidities	0.40 (0.38–0.42)	0.52 (0.48–0.57)	0.36 (0.34–0.38)	0.33 (0.30–0.35)	0.33 (0.31–0.34)	0.60 (0.56–0.64)
D + and 2 morbidities	1.42 (1.36–1.48)	1.78 (1.59–1.99)	1.36 (1.27–1.45)	1.09 (1.01–1.19)	1.17 (1.10–1.23)	1.90 (1.75–2.05)
D + and 3 + morbidities	4.26 (4.02–4.52)	5.44 (4.70–6.30)	3.95 (3.63–4.29)	3.32 (3.00–3.69)	3.36 (3.13–3.61)	5.91 (5.34–6.54)
**Hospitalization from home** ^**2**^						
D- and 0–1 morbidities	1	1	1	1	1	1
D- and 2 morbidities	2.65 (2.54–2.77)	2.78 (2.61–2.97)	2.67 (2.49–2.87)	2.17 (1.95–2.41)	2.37 (2.23–2.52)	2.97 (2.80–3.16)
D- and 3 + morbidities	6.50 (6.19–6.82)	7.75 (7.17–8.38)	6.10 (5.66–6.58)	4.72 (4.22–5.28)	5.43 (5.07–5.81)	7.81 (7.29–8.37)
D + and 0–1 morbidities	0.63 (0.60–0.67)	0.80 (0.72–0.89)	0.60 (0.56–0.64)	0.49 (0.45–0.54)	0.51 (0.47–0.54)	0.89 (0.82–0.97)
D + and 2 morbidities	1.58 (1.50–1.67)	2.18 (1.91–2.50)	1.51 (1.39–1.64)	1.15 (1.03–1.28)	1.30 (1.21–1.40)	1.99 (1.82–2.18)
D + and 3 + morbidities	3.66 (3.44–3.90)	6.05 (5.12–7.14)	3.53 (3.22–3.86)	2.35 (2.08–2.65)	2.87 (2.64–3.11)	4.89 (4.41–5.41)
**Hospitalization from LTC** ^**3**^						
D- and 0–1 morbidities	1	1	1	1	1	1
D- and 2 morbidities	1.39 (1.31–1.47)	1.41 (1.23–1.62)	1.37 (1.25–1.50)	1.40 (1.28–1.55)	1.41 (1.31–1.52)	1.34 (1.21–1.49)
D- and 3 + morbidities	1.92 (1.82–2.03)	1.96 (1.73–2.23)	1.89 (1.74–2.05)	2.00 (1.82–2.19)	1.97 (1.84–2.11)	1.84 (1.67–2.03)
D + and 0–1 morbidities	0.74 (0.70–0.78)	0.93 (0.80–1.07)	0.78 (0.72–0.85)	0.63 (0.57–0.69)	0.70 (0.65–0.75)	0.86 (0.78–0.96)
D + and 2 morbidities	1.63 (1.54–1.73)	1.63 (1.41–1.89)	1.77 (1.62–1.93)	1.44 (1.31–1.59)	1.59 (1.48–1.70)	1.71 (1.54–1.90)
D + and 3 + morbidities	2.29 (2.16–2.43)	2.25 (1.96–2.59)	2.32 (2.13–2.53)	2.24 (2.03–2.46)	2.30 (2.15–2.47)	2.25 (2.03–2.48)

^1^ including persons who have had at least one day at home or LTC

^2^ including persons who have had at least one day at home

^3^ including persons who have had at least one day at LTC

### Models adjusted for age except age groups

The number of hospitalizations were lower for those with dementia than those without in the whole group and also when age groups and genders were analyzed separately, but the differences were not statistically significant in all groups. However, the IRR for hospitalizations from home was higher among those with dementia for the whole study group and all the subgroups at all levels of comorbidity, including those with only 0–1 comorbidities. For hospitalizations from LTC, the IRR was somewhat lower for those with dementia than those without and similar number of morbidities but the differences were not statistically significant. Multimorbidity increased not only the likelihood of hospitalization but also the number of hospitalizations for both people with and without dementia (Table 4). The predicted probabilities of the number of hospitalizations from the negative binomial regression model are displayed in Fig. 1 (lower panel).

**Table 4:** Association between dementia and comorbidities with number of hospitalizations from home/LTC, from home, and from LTC (separated models for whole group, age groups, and gender)

	All	70–79	80–89	90+	Women	Men
	IRR (95% CI)	IRR (95% CI)	IRR (95% CI)	IRR (95% CI)	IRR (95% CI)	IRR (95% CI)
**Hospitalization from home or LTC** ^**1**^						
D- and 0–1 morbidities	1	1	1	1	1	1
D- and 2 morbidities	1.46 (1.44–1.48)	1.46 (1.43–1.49)	1.45 (1.42–1.48)	1.47 (1.43–1.52)	1.40 (1.37–1.42)	1.53 (1.50–1.56)
D- and 3 + morbidities	2.16 (2.13–2.18)	2.12 (2.08–2.17)	2.15 (2.11–2.19)	2.21 (2.15–2.28)	2.09 (2.05–2.12)	2.23 (2.19–2.27)
D + and 0–1 morbidities	0.60 (0.59–0.61)	0.67 (0.64–0.71)	0.61 (0.59–0.62)	0.54 (0.52–0.56)	0.53 (0.51–0.54)	0.79 (0.77–0.82)
D + and 2 morbidities	1.25 (1.23–1.28)	1.28 (1.23–1.33)	1.25 (1.22–1.29)	1.20 (1.16–1.25)	1.15 (1.12–1.18)	1.42 (1.38–1.46)
D + and 3 + morbidities	2.04 (2.01–2.07)	2.01 (1.95–2.08)	2.01 (1.97–2.06)	2.05 (1.98–2.12)	1.92 (1.88–1.96)	2.18 (2.12–2.23)
**Hospitalization from home** ^**2**^						
D- and 0–1 morbidities	1	1	1	1	1	1
D- and 2 morbidities	1.50 (1.47–1.52)	1.50 (1.47–1.54)	1.50 (1.47–1.54)	1.45 (1.40–1.50)	1.42 (1.40–1.45)	1.58 (1.55–1.61)
D- and 3 + morbidities	2.20 (2.17–2.23)	2.21 (2.16–2.26)	2.23 (2.18–2.27)	2.10 (2.04–2.17)	2.10 (2.07–2.14)	2.32 (2.27–2.36)
D + and 0–1 morbidities	1.39 (1.36–1.42)	1.40 (1.33–1.47)	1.40 (1.36–1.45)	1.31 (1.25–1.37)	1.29 (1.25–1.33)	1.52 (1.46–1.58)
D + and 2 morbidities	1.92 (1.88–1.96)	1.95 (1.86–2.03)	1.94 (1.88–1.99)	1.84 (1.76–1.91)	1.84 (1.79–1.89)	2.01 (1.95–2.08)
D + and 3 + morbidities	2.66 (2.61–2.70)	2.71 (2.62–2.81)	2.68 (2.62–2.75)	2.54 (2.45–2.63)	2.55 (2.49–2.61)	2.78 (2.71–2.85)
**Hospitalization from LTC** ^**3**^						
D- and 0–1 morbidities	1	1	1	1	1	1
D- and 2 morbidities	1.47 (1.40–1.54)	1.40 (1.26–1.57)	1.44 (1.35–1.55)	1.54 (1.43–1.66)	1.48 (1.40–1.57)	1.41 (1.30–1.53)
D- and 3 + morbidities	2.41 (2.31–2.52)	2.41 (2.18–2.67)	2.39 (2.24–2.55)	2.48 (2.32–2.66)	2.44 (2.32–2.57)	2.28 (2.12–2.46)
D + and 0–1 morbidities	0.60 (0.57–0.62)	0.67 (0.60–0.76)	0.60 (0.56–0.64)	0.56 (0.52–0.60)	0.58 (0.55–0.61)	0.65 (0.60–0.71)
D + and 2 morbidities	1.36 (1.30–1.42)	1.34 (1.20–1.51)	1.38 (1.29–1.48)	1.31 (1.22–1.40)	1.34 (1.27–1.41)	1.36 (1.25–1.47)
D + and 3 + morbidities	2.24 (2.15–2.34)	2.17 (1.95–2.42)	2.22 (2.08–2.37)	2.28 (2.13–2.45)	2.25 (2.14–2.37)	2.12 (1.96–2.28)

^1^ including persons who have had at least one day at home or LTC

^2^ including persons who have had at least one day at home

^3^ including persons who have had at least one day at LTC

Person-time at risk (i.e., days at home or LTC) is included as an offset variable in each model

### Change in hospitalizations between the study years

**Fig. 2:**
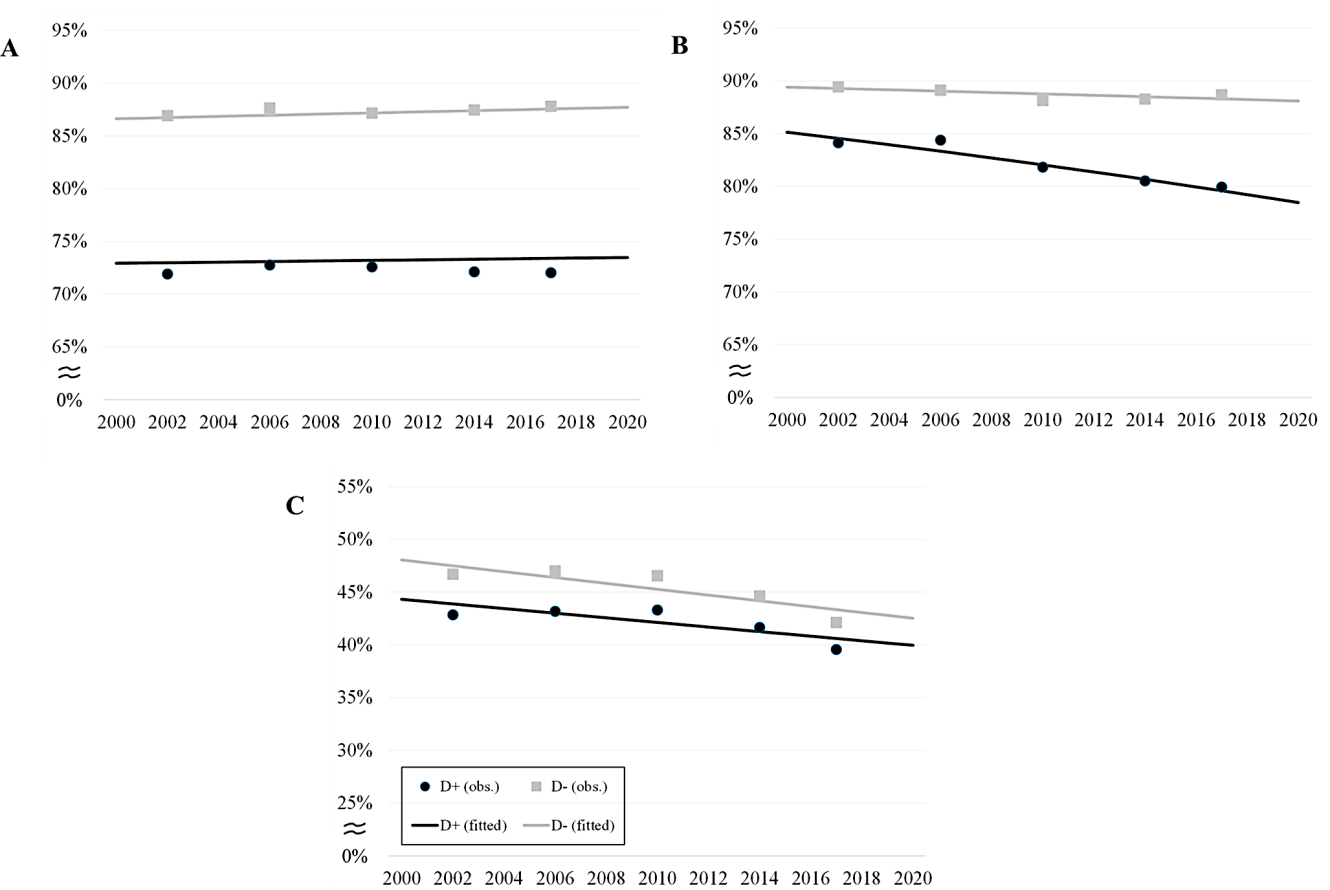
Observed and fitted probabilities of hospitalizations (based on logistic regression models) among people with and without dementia over the study years (**A**) from home or LTC, (**B**) from home, and (**C**) from LTC

Table 2 shows that the percentage of those who were hospitalized at least once during the last two years of life decreased between the study years especially in the younger age groups of people with dementia. In people without dementia, the decrease in hospitalization was clearest in hospitalizations from LTC. The percentage of those hospitalized from home or LTC decreased in both men and women. Hospitalizations for the whole group showed increasing trend (significant for those without dementia but non-significant for those with dementia) but there was no significant difference between the groups. Hospitalizations from home and LTC have declined over time in people with and without dementia. The decline in the hospitalizations from home was faster among those with dementia when compared to those without, and there was no difference between the groups for hospitalizations from LTC (Fig. 2- supplementary material).

## Discussion

Using data from comprehensive national registers spanning a 15-year period (2002–2017), this study adds to the limited evidence on the joint impact of dementia and comorbidities on hospitalizations in the last years of life. Multimorbidity increased hospitalizations among people with and without dementia: the greater the number of diagnoses, the higher the probability of hospitalization and the higher the number of hospitalizations in the last two years of life. In all, people with dementia had fewer hospitalizations than those without dementia. Overall, hospitalizations are relatively common during the last two years of life among both those with and without dementia: 8–9 out of ten men and women were hospitalized at least once, except women with dementia for whom the figure was only 6 to 7 out of ten.

Hospitalizations among people with dementia have been extensively explored, yet the findings on the role of comorbidity are scarce and somewhat contradictory. Most studies suggest that comorbidities increase the likelihood of hospitalization [8, 12, 23, 24], while a few studies have found no association or a somewhat elevated risk of hospitalization for people with dementia irrespective of other diseases [25, 26]. This variation depends on whether the study concentrates on the last stages of life and on national differences in health and social care service systems. Therefore, direct comparisons between different studies are difficult [23].

In this study that focuses on the last years of life, we found that people with dementia were less likely to be hospitalized and had fewer number of hospitalizations than those without at all levels of comorbidity. In both groups we observed a declining trend of hospitalization during the 15-year study period. These findings should be interpreted in the context of the health care and long-term care system. In Finland, all residents are covered by universal health care, which is funded by both taxes and user fees. Access to public health or long-term care is based on needs assessment, not on the individual’s ability to pay or private health care insurance. The total number of inpatient beds have decreased in the last decade in Finland. Round-the-clock LTC is mainly organized in the form of service housing with 24-hour assistance, which in recent decades has largely replaced traditional institutional settings, i.e., nursing homes and long-term hospital wards of health centers. Service housing is categorized as social care rather than health care, but residents also have access to basic health and medical care. National policy for the care of older people prioritizes living at home, which is supported by public home care. As a result, the proportion of older people living in care facilities has decreased while the share of those living at home has increased, even in advanced old age and among those with dementia. This increase of the proportion of old people living at home, from where the great majority of hospitalizations happen, is likely to explain the increasing trend of hospitalizations in the total study group [27–30].

The declining trend of hospitalization among people with dementia in the last years of life from both home and LTC may reflect the improved availability and skills of palliative and end-of-life care in LTC facilities [31]. However, we also observed this same trend for those living with dementia at home [32]. Among both men and women with and without dementia, hospitalizations from home were more frequent than hospitalizations from LTC, which likely reflects both the sufficiency of care received in LTC [25] and the present situation where people even with severe health problems live at home. However, as the hospitalizations from home were higher for individuals with dementia compared to those without dementia but similar number of comorbidities, it is important to identify individuals in this group that may benefit more from long term care.

Our study showed a clear difference in hospitalizations between people with and without dementia, but the reasons for the difference remain unclear. The type and severity of dementia and other diseases obviously influence the likelihood of hospitalization [33, 34], but our data are unable to shed light on that or on the immediate or underlying causes of hospitalizations. Basically, there are two opposite ways to interpret the differences. It is known that hospitalizations at the end of life are a burden to older individuals, particularly so for people with dementia who are vulnerable to changes in their environment and who may have difficulty understanding them [35]. Avoiding hospitalization, providing care at home or in a care home and perhaps a more passive treatment of comorbidities may be justified and in the interest of the person living with advanced dementia [36, 37]. On the other hand, the reduced use of hospital care among people with dementia may be due to poorer diagnostics and limited access to medical care resulting from their condition, even in cases where hospital treatment would be in the patient’s best interest and improve the quality of their remaining life.

We also found sex differences in hospitalization in the last two years of life, particularly among people with dementia. In each study year, hospitalizations from both home and LTC were more frequent among men than women. In people without dementia, we found virtually no differences. This is remarkable in that at least in very old age, when dementia is most frequent, women have more comorbidities than men [38]. We found no obvious reasons for this sex difference in our study. What we do know is that men retain their married status longer than women, who more often live as widows or single. It is possible that a spouse can be instrumental in organizing hospital care for an ailing husband [39].

The strengths of our study include its use of exhaustive nationwide register data on almost 190,000 individuals aged 70 + for a long study period, including both care records and death certificates for the diagnoses. The Finnish care registers and the causes of death register are considered reliable [40]. Although some errors may occur, as in all types of data, the Finnish health and care registers have been collected for decades which diminishes the possibility of structural errors. We did not have information on the stage of dementia or the immediate causes of hospitalizations. However, using several nationwide registers linked by personal identity numbers offers a unique possibility for reliable research and reduces the possibility of underestimating the frequency of dementia diagnosis, which could be possible if we used only care registers that are dependent on a person’s access to care [41]. We were able to assess several factors with potential impact on hospitalizations. Our extensive register-based data provides reliable and high-quality results, supporting the strength of our research.

## Conclusion

Dementia is a major age-related disease whose frequency is rising with increasing life expectancies, and it is a leading cause of need for care in old age. Individuals with dementia are vulnerable and their quality of life warrants special awareness. We found that at all levels of comorbidity people with dementia have a lower likelihood of hospitalization and a lower number of hospitalizations during the last years of life than people without dementia. Comorbidities were the main driver for hospitalizations in dementia. Hospitalization showed a declining trend in both groups during the 15-year study period. Therefore, it is critically important to determine whether the reduced use of hospital care among people with dementia is due to their lower needs or sufficient care elsewhere, or the result of poor and unequal access to medical care.

## Electronic supplementary material

Below is the link to the electronic supplementary material.

Supplementary Material 1

## Data Availability

No datasets were generated or analysed during the current study.

## Abbreviations

ICD: International classification of diseases
CAD: Coronary artery disease
LTC: Long term care
OR: Odds ratio
CI: Confidence interval
IRR: Incidence rate ratio
SD: Standard deviation

## References

[1] Maxwell CJ, Amuah JE, Hogan DB, Cepoiu-Martin M, Gruneir A, Patten SB et al (2015) Elevated hospitalization risk of assisted living residents with dementia in Alberta, Canada. J Am Med Dir Assoc 16(7):568–577. 10.1016/j.jamda.2015.01.079 25717011 10.1016/j.jamda.2015.01.079

[2] Allers K, Hoffmann F (2018) Mortality and hospitalization at the end of life in newly admitted nursing home residents with and without dementia. Soc Psychiatry Psychiatr Epidemiol 53(8):833–839. 10.1007/s00127-018-1523-0 29721593 10.1007/s00127-018-1523-0

[3] Reynish EL, Hapca SM, De Souza N, Cvoro V, Donnan PT, Guthrie B (2017) Epidemiology and outcomes of people with dementia, delirium, and unspecified cognitive impairment in the general hospital: prospective cohort study of 10,014 admissions. BMC Med 15(1):140. 10.1186/s12916-017-0899-0 28747225 10.1186/s12916-017-0899-0 PMC5530485

[4] Wolf D, Rhein C, Geschke K, Fellgiebel A (2019) Preventable hospitalizations among older patients with cognitive impairments and dementia. Int Psychogeriatr 31(3):383–391. 10.1017/S1041610218000960 30221613 10.1017/S1041610218000960

[5] Sakata N, Okumura Y, Fushimi K, Nakanishi M, Ogawa A (2018) Dementia and risk of 30-Day readmission in older adults after discharge from Acute Care hospitals. J Am Geriatr Soc 66(5):871–878. 10.1111/jgs.15282 29460284 10.1111/jgs.15282

[6] Bail K, Goss J, Draper B, Berry H, Karmel R, Gibson D (2015) The cost of hospital-acquired complications for older people with and without dementia; a retrospective cohort study. BMC Health Serv Res 15:91. 10.1186/s12913-015-0743-1 25890030 10.1186/s12913-015-0743-1 PMC4376999

[7] Aaltonen M, El Adam S, Martin-Matthews A, Sakamoto M, Strumpf E, McGrail K (2021) Dementia and poor continuity of primary care Delay Hospital Discharge in older adults: a Population-based study from 2001 to 2016. J Am Med Dir Assoc 22(7):1484–1492e3. 10.1016/j.jamda.2020.11.030 33358723 10.1016/j.jamda.2020.11.030

[8] Phelan EA, Borson S, Grothaus L, Balch S, Larson EB (2012) Association of incident dementia with hospitalizations. JAMA 307(2):165–172. 10.1001/jama.2011.1964 22235087 10.1001/jama.2011.1964 PMC3312921

[9] Weber SR, Pirraglia PA, Kunik ME (2011) Use of services by community-dwelling patients with dementia: a systematic review. Am J Alzheimers Dis Other Demen 26(3):195–204. 10.1177/1533317510392564 21273207 10.1177/1533317510392564 PMC10845557

[10] Fox C, Smith T, Maidment I, Hebding J, Madzima T, Cheater F et al (2014) The importance of detecting and managing comorbidities in people with dementia? Age Ageing 43(6):741–743. 10.1093/ageing/afu101 25038831 10.1093/ageing/afu101

[11] Zhao Y, Kuo TC, Weir S, Kramer MS, Ash AS (2008) Healthcare costs and utilization for Medicare beneficiaries with Alzheimer’s. BMC Health Serv Res 8:108. 10.1186/1472-6963-8-108 18498638 10.1186/1472-6963-8-108 PMC2424046

[12] Shepherd H, Livingston G, Chan J, Sommerlad A (2019) Hospitalisation rates and predictors in people with dementia: a systematic review and meta-analysis. BMC Med 17(1):130. 10.1186/s12916-019-1369-7 31303173 10.1186/s12916-019-1369-7 PMC6628507

[13] Gungabissoon U, Perera G, Galwey NW, Stewart R (2020) The association between dementia severity and hospitalisation profile in a newly assessed clinical cohort: the South London and Maudsley case register. BMJ Open 10(4):e035779. 10.1136/bmjopen-2019-035779 32284392 10.1136/bmjopen-2019-035779 PMC7200045

[14] Rosenwax L, McNamara B, Zilkens R (2009) A population-based retrospective cohort study comparing care for western australians with and without Alzheimer’s disease in the last year of life. Health Soc Care Community 17(1):36–44. 10.1111/j.1365-2524.2008.00795.x 18564194 10.1111/j.1365-2524.2008.00795.x

[15] McCormick WC, Hardy J, Kukull WA, Bowen JD, Teri L, Zitzer S et al (2001) Healthcare utilization and costs in managed care patients with Alzheimer’s disease during the last few years of life. J Am Geriatr Soc 49(9):1156–1160. 10.1046/j.1532-5415.2001.49231.x 11559373 10.1046/j.1532-5415.2001.49231.x

[16] Macdonald A, Cooper B (2007) Long-term care and dementia services: an impending crisis. Age Ageing 36(1):16–22. 10.1093/ageing/afl126 17175565 10.1093/ageing/afl126

[17] Hendriks SA, Smalbrugge M, Deliens L, Koopmans RTCM, Onwuteaka-Philipsen BD, Hertogh CMPM et al (2017) End-of-life treatment decisions in nursing home residents dying with dementia in the Netherlands. Int J Geriatr Psychiatry 32(12):e43–e49. 10.1002/gps.4650 28032354 10.1002/gps.4650

[18] Houttekier D, Vandervoort A, Van den Block L, van der Steen JT, Vander Stichele R, Deliens L (2014) Hospitalizations of nursing home residents with dementia in the last month of life: results from a nationwide survey. Palliat Med 28(9):1110–1117. 10.1177/0269216314535962 24866759 10.1177/0269216314535962

[19] Aaltonen MS, Forma LP, Pulkki JM, Raitanen JA, Rissanen P, Jylhä MK (2019) The joint impact of age at death and dementia on long-term care use in the last years of life: changes from 1996 to 2013 in Finland. Gerontol Geriatr Med 5:2333721419870629. 10.1177/2333721419870629 31489341 10.1177/2333721419870629 PMC6709434

[20] Bauer K, Schwarzkopf L, Graessel E, Holle R (2014) A claims data-based comparison of comorbidity in individuals with and without dementia. BMC Geriatr 14:10. 10.1186/1471-2318-14-10 24472217 10.1186/1471-2318-14-10 PMC3909381

[21] Poblador-Plou B, Calderón-Larrañaga A, Marta-Moreno J, Hancco-Saavedra J, Sicras-Mainar A, Soljak M et al (2014) Comorbidity of dementia: a cross-sectional study of primary care older patients. BMC Psychiatry 14:84. 10.1186/1471-244X-14-84 24645776 10.1186/1471-244X-14-84 PMC3994526

[22] Vogelgsang J, Wolff-Menzler C, Kis B, Abdel-Hamid M, Wiltfang J, Hessmann P (2018) Cardiovascular and metabolic comorbidities in patients with Alzheimer’s disease and vascular dementia compared to a psychiatric control cohort. Psychogeriatr off J Jpn Psychogeriatr Soc 18(5):393–401. 10.1111/psyg.12338 10.1111/psyg.12338 29993172

[23] Sommerlad A, Perera G, Mueller C, Singh-Manoux A, Lewis G, Stewart R et al (2019) Hospitalisation of people with dementia: evidence from English electronic health records from 2008 to 2016. Eur J Epidemiol 34(6):567–577. 10.1007/s10654-019-00481-x 30649705 10.1007/s10654-019-00481-x PMC6497615

[24] Lin PJ, Fillit HM, Cohen JT, Neumann PJ (2013) Potentially avoidable hospitalizations among Medicare beneficiaries with Alzheimer’s disease and related disorders. Alzheimers Dement J Alzheimers Assoc 9(1):30–38. 10.1016/j.jalz.2012.11.002 10.1016/j.jalz.2012.11.002 23305822

[25] Afonso-Argilés FJ, Meyer G, Stephan A, Comas M, Wübker A, Leino-Kilpi H et al (2020) Emergency department and hospital admissions among people with dementia living at home or in nursing homes: results of the European RightTimePlaceCare project on their frequency, associated factors and costs. BMC Geriatr 20(1):453. 10.1186/s12877-020-01835-x 33153444 10.1186/s12877-020-01835-x PMC7643440

[26] Bynum JPW, Rabins PV, Weller W, Niefeld M, Anderson GF, Wu AW (2004) The relationship between a dementia diagnosis, chronic illness, medicare expenditures, and hospital use. J Am Geriatr Soc 52(2):187–194. 10.1111/j.1532-5415.2004.52054.x 14728626 10.1111/j.1532-5415.2004.52054.x

[27] Ministry of Social Affairs and Health [Internet] [cited 2024 Aug 12]. Older people services. Available from: https://stm.fi/en/older-people-services

[28] National Programme on Ageing (2030): For an age-competent Finland [Internet]. Ministry of Social Affairs and Health; 2020 [cited 2023 Feb 7]. Available from: https://julkaisut.valtioneuvosto.fi/handle/10024/162596

[29] Tolppanen AM, Taipale H, Purmonen T, Koponen M, Soininen H, Hartikainen S (2015) Hospital admissions, outpatient visits and healthcare costs of community-dwellers with Alzheimer’s disease. Alzheimers Dement J Alzheimers Assoc 11(8):955–963. 10.1016/j.jalz.2014.10.005 10.1016/j.jalz.2014.10.005 25496872

[30] OECD/European Union (2022) Health at a glance: Europe 2022, state of Health in the EU cycle. OECD Publishing, Paris. 10.1787/507433b0-en

[31] Froggatt KA, Moore DC, Van den Block L, Ling J, Payne SA, Van den Block L et al (2020) Palliative Care implementation in Long-Term Care facilities: European Association for Palliative Care White Paper. J Am Med Dir Assoc 21(8):1051–1057. 10.1016/j.jamda.2020.01.009 32115370 10.1016/j.jamda.2020.01.009

[32] Niu H, Alvarez-Alvarez I, Aguinaga-Ontoso I, Guillen-Grima F (2018) Trends in Hospital Morbidity from Alzheimer’s Disease in the European Union, 2000 to 2014. Am J Alzheimers Dis Other Demen 33(7):440–449. 10.1177/1533317518787270 30068226 10.1177/1533317518787270 PMC10852452

[33] Lamb VL, Sloan FA, Nathan AS (2008) Dementia and Medicare at life’s end. Health Serv Res 43(2):714–732. 10.1111/j.1475-6773.2007.00787.x 18370975 10.1111/j.1475-6773.2007.00787.x PMC2442376

[34] Murman DL, Chen Q, Colucci PM, Colenda CC, Gelb DJ, Liang J Comparison of healthcare utilization and direct costs in three degenerative dementias. Am J Geriatr Psychiatry Off J Am Assoc Geriatr Psychiatry [Internet]. 2002 Jun [cited 2023 Feb 7];10(3). Available from: https://pubmed.ncbi.nlm.nih.gov/11994221/ (article can be found from here: 10.1097/00019442-200205000-00013) 11994221

[35] James BD, Wilson RS, Capuano AW, Boyle PA, Shah RC, Lamar M et al (2019) Hospitalization, Alzheimer’s Disease and related neuropathologies, and Cognitive decline. Ann Neurol 86(6):844–852. 10.1002/ana.25621 31614018 10.1002/ana.25621 PMC6973140

[36] Martinsson L, Lundström S, Sundelöf J (2020) Better quality of end-of-life care for persons with advanced dementia in nursing homes compared to hospitals: a Swedish national register study. BMC Palliat Care 19(1):135. 10.1186/s12904-020-00639-5 32847571 10.1186/s12904-020-00639-5 PMC7449048

[37] Yorganci E, Stewart R, Sampson EL, Sleeman KE (2022) Patterns of unplanned hospital admissions among people with dementia: from diagnosis to the end of life. Age Ageing 51(5):afac098. 10.1093/ageing/afac098 35581158 10.1093/ageing/afac098 PMC9113942

[38] Halonen P, Enroth L, Jämsen E, Vargese S, Jylhä M (2022) Dementia and related comorbidities in the Population aged 90 and over in the vitality 90 + study, Finland: patterns and trends from 2001 to 2018. J Aging Health 8982643221123451. 10.1177/08982643221123451 10.1177/08982643221123451 PMC10150268 36256914

[39] Wu-Chung EL, Leal SL, Denny BT, Cheng SL, Fagundes CP (2022) Spousal caregiving, widowhood, and cognition: a systematic review and a biopsychosocial framework for understanding the relationship between interpersonal losses and dementia risk in older adulthood. Neurosci Biobehav Rev 134:104487. 10.1016/j.neubiorev.2021.12.010 34971701 10.1016/j.neubiorev.2021.12.010 PMC8925984

[40] Sund R (2012) Quality of the Finnish Hospital Discharge Register: a systematic review. Scand J Public Health 40(6):505–515. 10.1177/1403494812456637 22899561 10.1177/1403494812456637

[41] Laugesen K, Ludvigsson JF, Schmidt M, Gissler M, Valdimarsdottir UA, Lunde A et al (2021) Nordic Health Registry-Based Research: a review of Health Care systems and Key registries. Clin Epidemiol 13:533–554. 10.2147/CLEP.S314959 34321928 10.2147/CLEP.S314959 PMC8302231

