# Supplementary figures and images for: Hospitalizations of the older adults with and without dementia during the last two years of life: the impact of comorbidity and changes from 2002 to 2017
Source: Aging Clin Exp Res. 2025 Jan 21;37(1):25. doi: 10.1007/s40520-024-02918-0 (PMC11753344; doi:10.1007/s40520-024-02918-0)

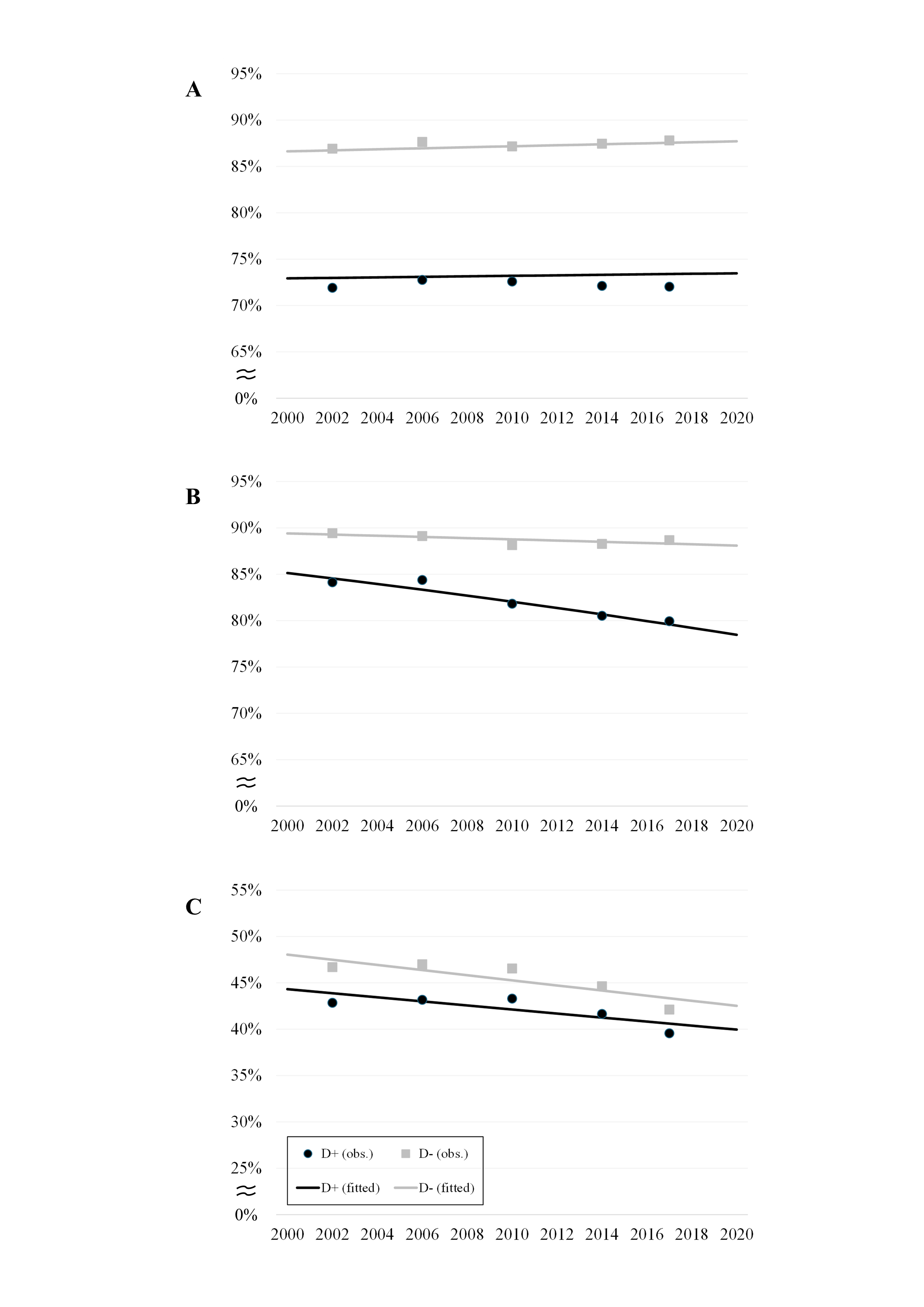

Supplement: Supplementary file 1 — Supplementary Material 1 [file 40520_2024_2918_MOESM1_ESM.tif]
